# Supplementary material for: OpenMM 7: Rapid development of high performance algorithms for molecular dynamics
Source: PLoS Comput Biol. 2017 Jul 26;13(7):e1005659. doi: 10.1371/journal.pcbi.1005659 (PMC5549999; doi:10.1371/journal.pcbi.1005659)
Supplement: S1 Source Code — (ZIP) [file pcbi.1005659.s002.zip › OpenMM-7.0.1-Source/docs/api-python/generated/simtk.openmm.openmm.AndersenThermostat.html]

AndersenThermostat — OpenMM 7.0.1.dev-5e86c4f documentation


# AndersenThermostat¶

*class* `simtk.openmm.openmm.``AndersenThermostat`(*\*args*)¶
:   This class uses the Andersen method to maintain constant temperature.

    `__init__`(*self*, *defaultTemperature*, *defaultCollisionFrequency*) → AndersenThermostat¶
    :   \_\_init\_\_(self, other) -> AndersenThermostat

        Create an AndersenThermostat.

        |  |  |
        | --- | --- |
        | Parameters: | - **defaultTemperature** (*double*) – the default temperature of the heat bath (in Kelvin) - **defaultCollisionFrequency** (*double*) – the default collision frequency (in 1/ps) |

    Methods

    |  |  |
    | --- | --- |
    | `CollisionFrequency`(() -> std::string const &) | This is the name of the parameter which store the current collision frequency (in 1/ps). |
    | `Temperature`(() -> std::string const &) | This is the name of the parameter which stores the current temperature of the heat bath (in Kelvin). |
    | `__init__`((self, defaultTemperature, ...) | \_\_init\_\_(self, other) -> AndersenThermostat |
    | `getDefaultCollisionFrequency`((self) -> double) | Get the default collision frequency (in 1/ps). |
    | `getDefaultTemperature`((self) -> double) | Get the default temperature of the heat bath (in Kelvin). |
    | `getForceGroup`((self) -> int) | Get the force group this Force belongs to. |
    | `getRandomNumberSeed`((self) -> int) | Get the random number seed. |
    | `setDefaultCollisionFrequency`(self, frequency) | Set the default collision frequency. |
    | `setDefaultTemperature`(self, temperature) | Set the default temperature of the heat bath. |
    | `setForceGroup`(self, group) | Set the force group this Force belongs to. |
    | `setRandomNumberSeed`(self, seed) | Set the random number seed. |
    | `usesPeriodicBoundaryConditions`((self) -> bool) | Returns whether or not this force makes use of periodic boundary conditions. |

    *static* `Temperature`() → std::string const &¶
    :   This is the name of the parameter which stores the current temperature of the heat bath (in Kelvin).

    *static* `CollisionFrequency`() → std::string const &¶
    :   This is the name of the parameter which store the current collision frequency (in 1/ps).

    `getDefaultTemperature`(*self*) → double¶
    :   Get the default temperature of the heat bath (in Kelvin).

        |  |  |
        | --- | --- |
        | Returns: | the default temperature of the heat bath, measured in Kelvin. |
        | Return type: | double |

    `setDefaultTemperature`(*self*, *temperature*)¶
    :   Set the default temperature of the heat bath. This will affect any new Contexts you create, but not ones that already exist.

        |  |  |
        | --- | --- |
        | Parameters: | **temperature** (*double*) – the default temperature of the heat bath (in Kelvin) |

    `getDefaultCollisionFrequency`(*self*) → double¶
    :   Get the default collision frequency (in 1/ps).

        |  |  |
        | --- | --- |
        | Returns: | the default collision frequency, measured in 1/ps. |
        | Return type: | double |

    `setDefaultCollisionFrequency`(*self*, *frequency*)¶
    :   Set the default collision frequency. This will affect any new Contexts you create, but not ones that already exist.

        |  |  |
        | --- | --- |
        | Parameters: | **frequency** (*double*) – the default collision frequency (in 1/ps) |

    `getRandomNumberSeed`(*self*) → int¶
    :   Get the random number seed. See setRandomNumberSeed() for details.

    `setRandomNumberSeed`(*self*, *seed*)¶
    :   Set the random number seed. The precise meaning of this parameter is undefined, and is left up to each Platform to interpret in an appropriate way. It is guaranteed that if two simulations are run with different random number seeds, the sequence of collisions will be different. On the other hand, no guarantees are made about the behavior of simulations that use the same seed. In particular, Platforms are permitted to use non-deterministic algorithms which produce different results on successive runs, even if those runs were initialized identically.

        If seed is set to 0 (which is the default value assigned), a unique seed is chosen when a Context is created from this Force. This is done to ensure that each Context receives unique random seeds without you needing to set them explicitly.

    `usesPeriodicBoundaryConditions`(*self*) → bool¶
    :   Returns whether or not this force makes use of periodic boundary conditions.

        |  |  |
        | --- | --- |
        | Returns: | true if force uses PBC and false otherwise |
        | Return type: | bool |

    `__delattr__`¶
    :   x.\_\_delattr\_\_(‘name’) <==> del x.name

    `__format__`()¶
    :   default object formatter

    `__getattribute__`¶
    :   x.\_\_getattribute\_\_(‘name’) <==> x.name

    `__hash__`¶

    `__reduce__`()¶
    :   helper for pickle

    `__reduce_ex__`()¶
    :   helper for pickle

    `__sizeof__`() → int¶
    :   size of object in memory, in bytes

    `__str__`¶

    `getForceGroup`(*self*) → int¶
    :   Get the force group this Force belongs to.

    `setForceGroup`(*self*, *group*)¶
    :   Set the force group this Force belongs to.

        |  |  |
        | --- | --- |
        | Parameters: | **group** (*int*) – the group index. Legal values are between 0 and 31 (inclusive). |

High performance molecular simulation on GPUs


### Navigation

- Application Layer
- Library Layer
  - Core Objects
  - Forces
  - Integrators
  - Extras

©2015, Stanford University and the Authors.
|
Powered by Sphinx 1.3.1
& Alabaster 0.7.7
|
Page source
